# Quantum tunneling and quantum walks as algorithmic resources to solve hard *K*-SAT instances

**DOI:** 10.1038/s41598-021-95801-1

**Published:** 2021-08-19

**Authors:** Ernesto Campos, Salvador E. Venegas-Andraca, Marco Lanzagorta

**Affiliations:** 1 Tecnologico de Monterrey, Escuela de Ingenieria y Ciencias, Av Eugenio Garza Sada 2501, Mty, NL Mexico; 2 Skolkovo Institute of Science and Technology, 3 Nobel Street, Skolkovo, 121205 Moscow Russia; 3 US Naval Research Laboratory, 4555 Overlook Ave., SW Washington, DC 20375 USA

**Keywords:** Computer science, Quantum information

## Abstract

We present a new quantum heuristic algorithm aimed at finding satisfying assignments for hard *K*-SAT instances using a continuous time quantum walk that explicitly exploits the properties of quantum tunneling. Our algorithm uses a Hamiltonian $H_A(F)$ which is specifically constructed to solve a *K*-SAT instance *F*. The heuristic algorithm aims at iteratively reducing the Hamming distance between an evolving state ${|{\psi _j}\rangle }$ and a state that represents a satisfying assignment for *F*. Each iteration consists on the evolution of ${|{\psi _j}\rangle }$ (where *j* is the iteration number) under $e^{-iH_At}$, a measurement that collapses the superposition, a check to see if the post-measurement state satisfies *F* and in the case it does not, an update to $H_A$ for the next iteration. Operator $H_A$ describes a continuous time quantum walk over a hypercube graph with potential barriers that makes an evolving state to scatter and mostly follow the shortest tunneling paths with the smaller potentials that lead to a state ${|{s}\rangle }$ that represents a satisfying assignment for *F*. The potential barriers in the Hamiltonian $H_A$ are constructed through a process that does not require any previous knowledge on the satisfying assignments for the instance *F*. Due to the topology of $H_A$ each iteration is expected to reduce the Hamming distance between each post measurement state and a state ${|{s}\rangle }$. If the state ${|{s}\rangle }$ is not measured after *n* iterations (the number *n* of logical variables in the instance *F* being solved), the algorithm is restarted. Periodic measurements and quantum tunneling also give the possibility of getting out of local minima. Our numerical simulations show a success rate of 0.66 on measuring ${|{s}\rangle }$ on the first run of the algorithm (i.e., without restarting after *n* iterations) on thousands of 3-SAT instances of 4, 6, and 10 variables with unique satisfying assignments.

## Introduction

*K*-SAT is a most important problem in computer science which is known to be $\mathsf {NP-Complete}$ for all $\text {K}>2$^1,2^. We focus on the set of satisfiable instances of the *K*-SAT, i.e. those instances for which there is at least one assignment that returns a logical 1. Satisfying a *K*-SAT instance may be easy or require very significant efforts, it all depends on its number of variables and clauses. Instances with one or few satisfactory assignments are harder to solve because satisfactory assignments live in a set with cardinality $2^n$, where *n* is the number of binary variables upon which the instance is built. Those *K*-SAT instances for which there is only one satisfying assignment constitute the focus of this paper.

A central activity in quantum computing consists of using quantum mechanical properties as resources in order to build algorithms that may outperform their classical counterparts. Along these lines, we find several quantum algorithms that have been designed to solve *K*-SAT instances^3–5^. In this paper, we present a quantum algorithm in which *continuous quantum walks and quantum tunneling are explicitly used as computational resources*.

Originally designed to model quantum phenomena^6–9^, quantum walks are an advanced tool for building quantum algorithms (e.g.^10–18^) that has been shown to constitute a universal model of quantum computation^19,20^. Quantum walks come in two variants: discrete and continuous^21^. The continuous-time quantum walk was proposed by Childs et al^11^ and the continuous time quantum walk on a hypercube was first studied by Kendon and Tregenna^22^ and later extensively analyzed by Drezgich et al^23^.

Quantum tunneling is a quantum mechanical phenomenon in which a particle passes through a potential barrier. The probability of finding a particle inside the potential barrier decreases exponentially, which in turn increases the probability of finding the particle outside the potential^24^, as if the particle were expelled from the barrier. This behavior suggests that quantum tunneling can be used as an algorithmic tool in which researchers can assign a meaning to the potential barriers in the context of the problem being solved. So far and to the best of our knowledge, quantum tunneling has only been used as a computational resource as a passive byproduct of quantum phenomena, i.e. as a physical phenomenon that comes out as a result of quantum evolution and that is utilized as a free variable upon which no explicit manipulation or control is performed. This is indeed the case of the most celebrated computational use of quantum tunneling as a component of quantum annealing^25,26^, and for alternative methods for hybrid search based on quantum annealing^27–29^. The closest to the use of tunneling in our algorithm is the work by Kechedzhi et al.^30^, where they use tunneling as a part of a quantum subroutine. Furthermore, the effects of potential barriers in discrete and continuous quantum walks in the quantum walk versions of the Grover’s algorithm were studied in^31^ but only as a type of noise that can be avoided, not as an algorithmic resource.

In this paper, we present a proposal for using quantum walks and quantum tunneling together, under explicit manipulation, in a quantum heuristic algorithm aimed at solving hard instances of the *K*-SAT problem. Our algorithm can deal with instances with multiple satisfying assignments but we have chosen to focus on instances with a unique satisfying assignment as instances with a few or unique satisfying assignments tend to be harder and the most important. For our testing we randomly generated, using a uniform distribution, thousands of 3-SAT instances of 4, 6, and 10 variables with one satisfying assignment. Such instances can be found in our repository^32^. We do not make any claim regarding computational speed-up. Our only claim is, based on the algorithm, theoretical results and simulations & experiments run on digital computers, that the use of quantum tunneling and quantum walks to solve *K*-SAT instances with unique solution is a promising approach.

The order in this paper is the following. The second section explains the “K-SAT problem”, “Continuous time quantum walk on a hypercube graph” shows the behavior of a quantum walk in a hyper cube graph, “Formal description of our algorithm” presents a formal description of the algorithm, its parameters, and a more complex example, “Implementation details” presents details on the algorithm implementation, the sixth section presents the “Analytical approximation of the algorithm behavior”, “Numerical calculation of parameters and additional simulations” presents numerical simulations and the calculation of the parameters of $H_A$, and “Conclusions” presents conclusions, observations and further work.

## K-SAT problem

The *K*-SAT problem is one of the most important problems in computer science. It is derived from the SAT problem, the first problem proved to be $\mathsf {NP-complete}$^1^. *K*-SAT is a constraint satisfaction problem where a Boolean formula has to be satisfied, i.e. its result has to be true. A *K*-SAT instance is written in conjunctive normal form (CNF), that is, a conjunction of clauses, where a clause is a disjunction of literals, and a literal can be a logical variable or its negation.

Formally speaking, the *K*-SAT problem can be stated as follows: given a *K*-SAT instance *P*, is there an satisfying truth assignment for *P*? In other words, the *K*-SAT problem consists of finding assignments that satisfy a given CNF Boolean formula, where *K* is the number of literals in each clause. For example:

$$\begin{aligned} P({\mathbf {x}})=\left( x_1\vee \overline{x}_3\vee \overline{x}_5\right) \wedge \left( x_2\vee \overline{x}_3\vee \overline{x}_6 \right) \wedge \left( x_1\vee \overline{x}_4\vee x_6 \right) \end{aligned}$$

where $\vee$ is the logical symbol for OR, $\wedge$ is the logical symbol for AND, $\{x_1, \ldots x_6\}$ is the set of logical binary variables, and $\overline{x}_j$ is the negation of $x_j$. This is a 3-SAT instance since each clause contains exactly 3 variables, and the binary string ${\mathbf {x}}_s=[1,1,0,0,1,0]$ is one of the assignments that satisfy $P({\mathbf {x}})$. This can be verified by substituting ${\mathbf {x}}_s$ into $P({\mathbf {x}})$

$$\begin{aligned} P({\mathbf {x}}_s)=\left( 1\vee 1\vee 0\right) \wedge \left( 1\vee 1\vee 1 \right) \wedge \left( 1\vee 1\vee 0 \right) =1 \end{aligned}$$

Satisfying a *K*-SAT instance *P* can be easy, difficult or impossible, depending on the number of bit strings that make *P* true. For example, let us suppose we have a *K*-SAT instance *P* defined over *n* binary variables. If we run a brute-force approach on *P*, we may simply substitute each and every bit string from ${\mathbf {x}}_0 = 000 \ldots 0$ to ${\mathbf {x}}_{2^n-1} = 111 \ldots 1$ on *P*, only stopping either when finding $P({\mathbf {x}}_s) = 1$ or when we have substituted all bit strings from ${\mathbf {x}}_0$ to ${\mathbf {x}}_{2^n-1}$ without finding any satisfying assignment. If *P* has *many* satisfying assignments, we will eventually find one of those assignments, possibly sooner than later. However, if *P* has only a few satisfying assignments (along these lines, the worst case scenario would be having only one satisfying assignment), finding them will eventually happen but it may well take a substantial (i.e. exponential) amount of time. If no bit string from ${\mathbf {x}}_0$ to ${\mathbf {x}}_{2^n-1}$ satisfies *P*, then *P* is unsatisfiable but it took us an exponential amount of resources to find that out.

As stated above, the K-SAT is an element of the set of $\mathsf {NP-Complete}$ problems, which in turn is a subset of $\mathsf {NP}$ (the abbreviation of “Non-deterministic Polynomial Time”), the set of problems in which a solution can be tested in polynomial time. Being an $\mathsf {NP-Complete}$ problem means that an arbitrary instance of any problem under the $\mathsf {NP}$ category can be rewritten as an instance of an $\mathsf {NP-Complete}$ problem in a polynomial number of steps^2,33,34^. The first problem proved to be $\mathsf {NP-Complete}$ was the SAT problem, and later the K-SAT, which is a variation of SAT, was proven to be also $\mathsf {NP-Complete}$ for $\text {K}>2$^1,2,34–36^. If an algorithm that runs on a Deterministic Turing Machine in polynomial time is discovered for an $\mathsf {NP-Complete}$ problem, it implies every $\mathsf {NP}$ problem can be solved in polynomial time, hence proving $\mathsf {P}=\mathsf {NP}$.

## Continuous time quantum walk on a hypercube graph

An *n*-dimensional hypercube graph can be constructed by assigning each vertex to one of the $2^n$ possible *n*-variable binary combinations, and connecting the nodes that have a distance of 1 in the Hamming distance (the number of positions/bits at which the corresponding elements between two arrays are different). The Hamiltonian $H_A$ used in our algorithm describes the dynamics of a quantum walk over a hypercube with potential barriers. The reason why we chose to use the hypercube graph is to reduce the distance between ${|{\psi _j}\rangle }$ and ${|{s}\rangle }$ with every iteration of the algorithm by taking advantage of the hypercube topology, where closer states have a shorter Hamming distance. During the evolution of an *n*-qubit state ${|{\psi _j}\rangle }$ (*j* is the iteration number) under $e^{-iH_At}$, most of the probability flux will tunnel through the paths from ${|{\psi _j}\rangle }$ to ${|{s}\rangle }$, and after a time $t_f$ the system is measured and the superposition collapses. If the post-measurement state ${|{\psi _{j+1}}\rangle }$ is different from ${|{s}\rangle }$, there is a good chance ${|{\psi _{j+1}}\rangle }$ is a state in a tunneling path from ${|{\psi _j}\rangle }$ and ${|{s}\rangle }$ which thanks to the hypercube topology will be at shorter Hamming distance to ${|{s}\rangle }$, $h_H({|{\psi _{j+1}}\rangle },{|{s}\rangle })<h_H({|{\psi _{j}}\rangle },{|{s}\rangle })$.

The structure of the the adjacency matrix *A*(*n*) for an *n*-dimensional hypercube can be written as

1 $$\begin{aligned} {A(n)=\sum _{j=1}^{n}A_j} \end{aligned}$$

where $${A_j}=\bigotimes ^{n}_{i=1}O_{ij}$$ and $$ O_{ij}= {\left\{ \begin{array}{ll} \hat{\sigma }_x &{}\text { if } i=j\\ I &{}\text { if } {i\ne j} \end{array}\right. } $$

If a state that is the tensor product of *n* qubits in the computational basis evolves under the unitary operator $e^{-iA(n)t}$ (which is equivalent to evolving under $H_A$ without potential barriers), the probability at some time of measuring a state ${|{z}\rangle }$, that is also an *n*-qubit tensor product, can be calculated as follows. Firstly, we need to realize that the Hermitian matrices $A_i$ commute with each other, for example

$$ \begin{aligned}   [A_{1} ,A_{3} ] =  & (\hat{\sigma }_{x}  \otimes I \otimes I \otimes ...)(I \otimes I \otimes \hat{\sigma }_{x}  \otimes ...) - (I \otimes I \otimes \hat{\sigma }_{x}  \otimes ...)(\hat{\sigma }_{x}  \otimes I \otimes I \otimes ...) \\    {\text{ = }} & (\hat{\sigma }_{{\text{x}}}  \otimes {\text{I}} \otimes \hat{\sigma }_{{\text{x}}}  \otimes ...) - (\hat{\sigma }_{{\text{x}}}  \otimes {\text{I}} \otimes \hat{\sigma }_{{\text{x}}}  \otimes ...) \\    [{\text{A}}_{{\text{1}}} ,{\text{A}}_{{\text{3}}} ]{\text{ = }} & {\text{0}} \\  \end{aligned}    $$

which means we can express $e^{-iA(n)t}$ as a product of unitary operators $e^{-iA(n)t}=e^{-iA_1t}e^{-iA_2t}...e^{-iA_nt}$. Then, by expanding ${e^{-iA_jt}}$ into a Taylor series and gathering the terms, we obtain

$$  \begin{aligned}   e^{{ - iA_{j} t}}  &  = I^{{ \otimes n}} \cos (t) - iA_{j} \sin (t) \\     &  = I^{{ \otimes n}} \cos (t) - i(I \otimes ... \otimes \hat{\sigma }_{x}  \otimes ... \otimes I)\sin (t) \\     &  = I \otimes ... \otimes (I\cos (t) - i\hat{\sigma }_{x} \sin (t)) \otimes ... \otimes I \\    e^{{ - iA_{j} t}}  &  = I \otimes ... \otimes e^{{ - i\hat{\sigma }_{x} t}}  \otimes ... \otimes I \\  \end{aligned}    $$

which means ${e^{-iA_jt}}$ is equivalent to only evolving the *i*th qubit under $e^{-i\hat{\sigma }_xt}$. As a result, we get $e^{-iA(n)t}=e^{-iA_1t}e^{-iA_2t}...e^{-iA_nt}={\bigotimes _{j=1}^{n}e^{-i\hat{\sigma }_x t}}$.

The probability $P_z(t)$ of measuring a state ${|{z}\rangle }$ from ${|{x}\rangle }$ evolving under $e^{-iA(n)t}$ depends only on the Hamming distance between ${|{x}\rangle }$ and ${|{z}\rangle }$. If we evolve ${|{x}\rangle }={|{0}\rangle }^{\otimes n}$, the probability of finding an arbitrary state ${|{z}\rangle }$ is given by

2 $$\begin{aligned} P_z(t)&=|{\langle {z}|}e^{-iA(n)t}{|{x}\rangle }|^2\nonumber \\&={\langle {z}|}\left( \bigotimes _{j=1}^{n}e^{-i\hat{\sigma }_x t}\right) {|{x}\rangle }{\langle {x}|}\left( \bigotimes _{j=1}^{n}e^{i\hat{\sigma }_x t}\right) {|{z}\rangle }\nonumber \\&={\langle {z}|}\bigotimes _{j=1}^{n}\left( e^{-i\hat{\sigma }_x t}{|{0}\rangle }{\langle {0}|}e^{i\hat{\sigma }_x t}\right) {|{z}\rangle }\nonumber \\&{={\langle {z}|}\bigotimes _{j=1}^{n}(\cos (t){|{0}\rangle }-i\sin (t){|{1}\rangle })(\cos (t){\langle {0}|}+i\sin (t){\langle {1}|}){|{z}\rangle }}\nonumber \\&=\cos ^{2n_0}(t)\sin ^{2n_1}(t) \end{aligned}$$

where $n_0$ and $n_1$ are the number of 0’s and 1s appearing in ${|{z}\rangle }$ respectively, meaning $n_1$ is also the Hamming distance between ${|{x}\rangle }$ and ${|{z}\rangle }$.
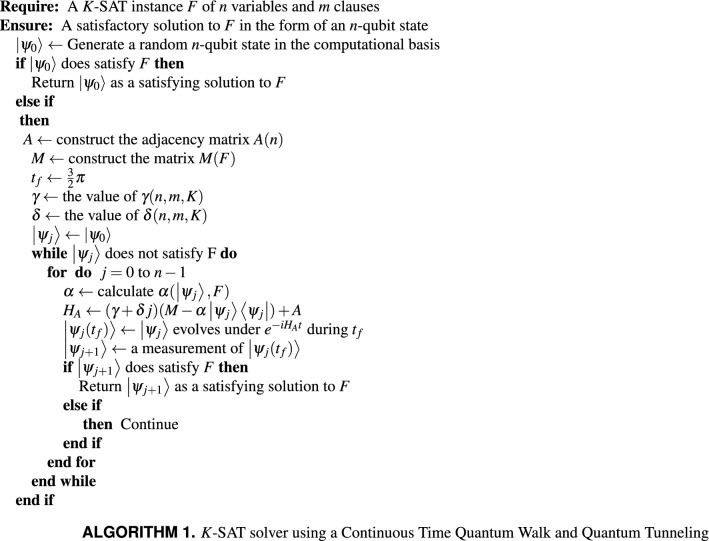


## Formal description of our algorithm

Our algorithm is formally introduced in ALGORITHM 1. A key element of our algorithm is the construction of the Hamiltonian $H_A(F,{|{\psi _j}\rangle },j)$, which determines the dynamics under which a state ${|{\psi _j}\rangle }$ will evolve during the *j*th iteration of the algorithm. Each iteration of the algorithm consists of the evolution of a state ${|{\psi _j}\rangle }$ during a time $t_f$ (this is the measurement frequency, for the cases analyzed in this paper $t_f=\frac{3}{2}\pi$ as discussed in “Numerical calculation of parameters and additional simulations”) followed by a measurement that collapses the superposition, and then a check of the post measurement state ${|{\psi _{j+1}}\rangle }$ to see if it represents a satisfying assignment for *F* (the checking can be done in *O*(*mK*)), in case it does not, $H_A$ gets updated for the next iteration.

The Hamiltonian $H_A$ for the *j*th iteration is

3 $$\begin{aligned} H_A(F,{|{\psi _j}\rangle },j)=A(n)+(\gamma +\delta j)(M-\alpha {|{\psi _j}\rangle }{\langle {\psi _j}|}) \end{aligned}$$

where *A*(*n*) is the adjacency matrix of an *n*-dimensional hypercube graph and *n* is the number of variables in the instance *F*, which can be constructed using Eq. (1), and

4 $$\begin{aligned} V=(\gamma +\delta j)(M-\alpha {|{\psi _j}\rangle }{\langle {\psi _j}|}) \end{aligned}$$

is the potential part of $H_A$. The components of *V* are:

- ${|{\psi _j}\rangle }$ is the tensor product of *n* qubits in the computational basis. ${|{\psi _j}\rangle }$ represents an assignment $\psi _j$ for the instance *F*. ${|{\psi _0}\rangle }$ is an initial random state, while ${|{\psi _j}\rangle }$ for $j>0$ is the post measurement state for the *j*th iteration.
- $\alpha = \alpha (F,\psi _j)$ is the number of clauses in *F* that are not satisfied by the assignment represented by ${|{\psi _j}\rangle }$.
- $M=M(F)$ is a diagonal matrix that has as the element corresponding to ${|{a}\rangle }{\langle {a}|}$ the number of clauses in *F* not satisfied by the assignment represented by ${|{a}\rangle }$. It is worth noting that the elements of *M*(*F*) arise naturally from a construction process (shown later in this section) without any prior knowledge on the satisfying solutions of *F*.
- $\gamma (n,m,K)$ and $\delta (n,m,K)$ are both positive real parameters dependent in the number of variables *n*, the number of clauses *m*, and the number of literals per clause *K* in the instance *F*. The reason of their dependency on *n*, *m*, and *K* is shown later in this section. $\gamma$ increases an amount $\delta$ every iteration which, as we explain later in this section, helps with the amplification of the state ${|{s}\rangle }$ that represents a satisfying assignment for *F* for an evolution during a time $t_f$ (the measurement frequency). We do not have an analytical expression for $\gamma$ and $\delta$, so we have numerically estimated their values for some cases. The values and their effects are discussed in more detail in “Effective Hamiltonian via degenerate perturbation theory” and “Numerical calculation of parameters and additional simulations”.

The rationale behind the elements of $H_A$ is the following. The potential *V* of Hamiltonian $H_A$ is a diagonal matrix with potential values specific to each state. The potential barriers between the states are designed to increase the tunneling between states ${|{\psi _j}\rangle }$ and ${|{s}\rangle }$. The reason most of the tunneling occurs between ${|{\psi _j}\rangle }$ and ${|{s}\rangle }$ is because the elements in the potential *V* corresponding to the outer products ${|{\psi _j}\rangle }{\langle {\psi _j}|}$ and ${|{s}\rangle }{\langle {s}|}$ are the only ones with a value of 0, which is a consequence of the construction process of *V*.

To construct the potential *V*, the first step is to build the Non-Satisfied Clauses matrix *M*(*F*). As stated before, *M*(*F*) is a diagonal matrix whose non-zero entries are the number of clauses in *F* which an assignment *a* does not satisfy. Assignment *a* is represented by state ${|{a}\rangle }$ and its corresponding non-zero entry in *M*(*F*) is located on the position that corresponds to the outer product ${|{a}\rangle }{\langle {a}|}$.

To show how *M*(*F*) is built, we now introduce an example based on $F_1({\mathbf {x}})=(x_1 \vee x_2) \wedge (x_1 \vee \overline{x}_2) \wedge (\overline{x}_1 \vee x_2) \wedge (\overline{x}_2 \vee x_3)$. The first clause in $F_1({\mathbf {x}})$ is $C_1=( x_1 \vee x_2)$, and the assignments that do not satisfy $C_1$ are : $x_1=0$, $x_2=0$, $x_3=0$, and $x_1=0$, $x_2=0$, $x_3=1$. The states that represent the assignments that do not satisfy $C_1$ are ${|{000}\rangle }$ and ${|{001}\rangle }$, and the sum of their outer products is $NS(C_1)={|{0}\rangle }{\langle {0}|} \otimes {|{0}\rangle }{\langle {0}|} \otimes {|{0}\rangle } {\langle {0}|}+{|{0}\rangle }{\langle {0}|} \otimes {|{0}\rangle }{\langle {0}|}\otimes {|{1}\rangle }{\langle {1}|}$, i.e.

5 $$\begin{aligned} NS(C_1)={|{0}\rangle }{\langle {0}|} \otimes {|{0}\rangle } {\langle {0}|}\otimes I \end{aligned}$$

The $NS(C_1)$ matrix form is given by ${\text {diag}}({1,1,0,0,0,0,0,0})$. Note that we can use *I* when doing the outer product summation to represent the variables that do not appear in a clause ($x_3$ in the case of $C_1$), hence reducing the number of elements needed to construct each $NS(C_i)$. The number of non *I* elements in every $NS(C_i)$ is only dependent on *K*, the number of binary variables on each and every clause of a *K*-SAT instance, which is fixed for a *K*-SAT instance. This allows us to construct each $NS(C_i)$ with a non-exponential number of elements (see “Analytical approximation of the algorithm behavior” for a detailed explanation of how to efficiently construct $NS(C_i)$ matrices). $NS(C_1)$ is a diagonal matrix with 1s in the elements corresponding to the outer products of the states that do not satisfy $C_1$. In this case the 1s are clearly in the positions corresponding to the outer products of ${|{000}\rangle }$ and ${|{001}\rangle }$, while the zeroes correspond to the outer products of the states that represent a satisfying assignment for $C_1$.

If the matrices $NS(C_i)$ are calculated for every clause in an instance *F*, the diagonal elements that are 0 in every $NS(C_i)$ correspond to the outer products of the states that satisfy every clause, thus satisfying instance *F*. The sum of every $NS(C_i)$ results in the diagonal matrix

6 $$\begin{aligned} M(F)=\sum _{i=1}^{m}NS(C_i), \end{aligned}$$

where an entry in *M*(*F*) corresponding to an outer product ${|{a}\rangle }{\langle {a}|}$ is equal to the number of clauses not satisfied in *F* by the assignment represented by ${|{a}\rangle }$, and *m* is the number of clauses in *F*. For our example $M(F_1)$ is

$$\begin{aligned} M(F_1) = {|{0}\rangle }{\langle {0}|} \otimes {|{0}\rangle } {\langle {0}|}\otimes I+{|{0}\rangle }{\langle {0}|} \otimes {|{1}\rangle } {\langle {1}|}\otimes I + {|{1}\rangle }{\langle {1}|} \otimes {|{0}\rangle } {\langle {0}|}\otimes I+I\otimes {|{1}\rangle }{\langle {1}|}\otimes {|{0}\rangle }{\langle {0}|} \end{aligned}$$

which in matrix notation is given by

7 $$\begin{aligned} {\text {diag}}({1,1,2,1,1,1,1,0}) \end{aligned}$$

From Eq. (7) we can see that the assignment corresponding to ${|{000}\rangle }$ does not satisfy 1 clause in $F_1$, ${|{001}\rangle }$ does not satisfy 1 clause, ${|{010}\rangle }$ does not satisfy 2 clauses, and so on. There is only one zero in the diagonal of $M(F_1)$, which corresponds to the outer product of ${|{111}\rangle }$, meaning the assignment $x_1=1$, $x_2=1$, $x_3=1$ is the only satisfying assignment for $F_1$.

Since the probability of tunneling is higher between states at potentials with similar values, we want to start with an initial state in a very small potential, expecting its probability flux to mostly tunnel through the shortest tunneling paths with the smaller potentials leading to ${|{s}\rangle }$ that represent a satisfying assignment for an instance *F* (the element corresponding to ${|{s}\rangle }{\langle {s}|}$ in *M*(*F*) is 0, the global minimum) thus amplifying the probability of measuring ${|{s}\rangle }$. Since we do not know a priori which states correspond to a small potential, we can just set to 0 the potential corresponding to the outer product ${|{\psi _j}\rangle }{\langle {\psi _j}|}$ by classically calculating the number of clauses $\alpha (\psi _j,F)$ the assignment corresponding to ${|{\psi _j}\rangle }$ does not satisfy (this can be done in *O*(*mK*)), and then subtracting the outer product ${|{\psi _j}\rangle }{\langle {\psi _j}|}$ multiplied by $\alpha (\psi _j,F)$ from the matrix *M*(*F*). As a result we get a 0 in the element corresponding to ${|{\psi _j}\rangle }{\langle {\psi _j}|}$ in *M*(*F*).

From Eq. (3), the Hamiltonian $H_A$ is

$$\begin{aligned} H_A(F,{|{\psi _j}\rangle },j)=A(n)+(\gamma +\delta j)(M-\alpha {|{\psi _j}\rangle }{\langle {\psi _j}|}) \end{aligned}$$

Suppose ${|{\psi _j}\rangle }={|{000}\rangle }$. We build *A*(*n*) using Eq. (1). Then, the resulting $H_A$ is given by

8 $$\begin{aligned} H_A(F_1,{|{000}\rangle },j)= \left[ {\begin{array}{llllllll} 0 &{} 1 &{} 1 &{} 0 &{} 1 &{} 0 &{} 0 &{} 0 \\ 1 &{} \gamma +\delta j &{} 0 &{} 1 &{} 0 &{} 1 &{} 0 &{} 0 \\ 1 &{} 0 &{} 2(\gamma +\delta j) &{} 1 &{} 0 &{} 0 &{} 1 &{} 0 \\ 0 &{} 1 &{} 1 &{} \gamma +\delta j &{} 0 &{} 0 &{} 0 &{} 1 \\ 1 &{} 0 &{} 0 &{} 0 &{} \gamma +\delta j &{} 1 &{} 1 &{} 0 \\ 0 &{} 1 &{} 0 &{} 0 &{} 1 &{} \gamma +\delta j &{} 0 &{} 1 \\ 0 &{} 0 &{} 1 &{} 0 &{} 1 &{} 0 &{} \gamma +\delta j &{} 1 \\ 0 &{} 0 &{} 0 &{} 1 &{} 0 &{} 1 &{} 1 &{} 0 \end{array}}\right] \end{aligned}$$

Note that explicitly calculating every element in M(F) consumes an exponential amount of resources in the number of qubits. We do it explicitly in this paper with the purpose of showing the logic and behavior of the algorithm. In a real world implementation, *M*(*F*) would be a physical process that acts as a blackbox.

### Parameters discussion

As stated before, $\gamma$ and $\delta$ are both real positive parameters. First we will explain the effect $\gamma +\delta j$ has in the probability of measuring ${|{s}\rangle }$, then we proceed to explain why it increases with every iteration, and finally why they are dependent on *n*, *m* and *K*, the number of variables, clauses, and literals per clause in *F* respectively.

- The effect $\gamma +\delta j$ has on $H_A$ is clearly a change in the size of the potential barriers. If $(\gamma +\delta j)<1$ the size of the potential barriers get reduced which makes a state ${|{\psi _j}\rangle }$ evolving under $e^{-iH_At}$ to tunnel and amplify ${|{s}\rangle }$ faster than if $(\gamma +\delta j)=1$. This occurs because the smaller the potential barriers the easier it is for the probability flux to transmit through them, but it has the unwanted side effect of letting more of the probability flux to transmit to states outside the shortest paths from ${|{\psi _j}\rangle }$ to ${|{s}\rangle }$ which translates into a smaller amplification of ${|{s}\rangle }$. On the other hand, if $(\gamma +\delta j)>1$ the time it will take ${|{\psi _j}\rangle }$ to tunnel to ${|{s}\rangle }$ increases but has the benefit of less probability flux getting out of the main tunneling paths from ${|{\psi _j}\rangle }$ to ${|{s}\rangle }$ which in turn increases the amplification and also reduces the small oscillations created by the reflections in the potentials and the probability flux transmitting out of the main tunneling paths. Simulations and analytical approximations showing the previously mentioned effects are shown in “Numerical calculation of parameters and additional simulations”.
- To understand why parameter $\gamma$ gets an increment $\delta$ on every iteration, it is required to understand how the time it takes to get the maximum amplification of ${|{s}\rangle }$ varies depending on its Hamming distance to ${|{\psi _j}\rangle }$, $d_H({|{s}\rangle },{|{\psi _j}\rangle })$. Intuitively, a state ${|{\psi _j}\rangle }$ evolving under $e^{-iH_At}$ will amplify faster ${|{s}\rangle }$ the smaller $d_H({|{\psi _j}\rangle },{|{s}\rangle })$ is, since it has to tunnel through less potential barriers (see “Numerical calculation of parameters and additional simulations” for simulations and approximations exhibiting this behavior). As explained above, thanks to the hypercube topology we have a good probability of getting a post measurement state ${|{\psi _{j+1}}\rangle }$ from the tunneling paths from ${|{\psi _j}\rangle }$ to ${|{s}\rangle }$ with a smaller Hamming distance to ${|{s}\rangle }$ compared to ${|{\psi _j}\rangle }$, $d_H({|{\psi _{j+1}}\rangle },{|{s}\rangle })<d_H({|{\psi _{j}}\rangle },{|{s}\rangle })$. So, the purpose of increments $\delta$ to $\gamma$ on every iteration is to keep approximately the same value for the measurement frequency $t_f$, so that the probability $P_s(t)$ of measuring ${|{s}\rangle }$ is greatly amplified for multiple distances $d_H({|{\psi _j}\rangle },{|{s}\rangle })$ approximately at the same time.
- Parameters $\gamma$, $\delta$ depend on *n*, *m* and *K* (the number of variables, clauses and literals per clause of *F* respectively) because as *n* increases, the average number of iterations also increases. This is because the average Hamming distance is also increased, and having greater $\gamma$ and $\delta$ can make tunneling too difficult from longer Hamming distances in few iterations. As for the dependency on *m* and *K* it is because we cannot know the value of every region in the potential *V* since they are $2^n$ values, but the average potential value $apv=\frac{m}{2^K}$ is only dependent on *m* and *K* for $(\gamma +\delta j)=1$ (the derivation of *apv* is presented in “Numerical calculation of parameters and additional simulations”, Eq. (11)). If an instance $F_1$ has a greater *apv* than an instance $F_2$ , and both instances have the same number of variables, we can expect the optimal values of $\gamma$ and $\delta$ for $F_1$ to be smaller than the optimal values for $F_2$ since smaller values of $\gamma$ and $\delta$ will facilitate the tunneling through the bigger potential barriers found in $H_A(F_1)$. We have estimated, via digital computer algorithms, optimal values of $\gamma$ and $\delta$, by doing a sweep over the values, for 3-SAT instances with 4, 6 and 10 variables with 16, 24, and 40 clauses respectively. Table 1 shows smaller values for larger instances with more variables in accordance to results presented above. The details on the numerical approximations of $\gamma$ and $\delta$ are shown in “Conclusions”.

**Table 1:** Optimal values for $\gamma$ and $\delta$ for various cases.

*K*	*n*	*m*	$\gamma$	$\delta$
3	4	16	2.6	0.5
3	6	24	1	0.6
3	10	40	1	0.45

We have also numerically estimated, for different values of $\gamma$ and $\delta$, the time during which we evolve the system $t_f$, the measurement frequency, giving the same value $t_f=4.72$ in every case (details in “Conclusions”). The analytical approximation of $t_f$ via perturbation theory (shown in “Numerical calculation of parameters and additional simulations”) gave $t_f=\frac{3}{2}\pi$, in close accordance with the numerical results.

## Implementation details

The algorithm may be efficiently implemented by approximating the evolution operator as a set of quantum gates by using techniques like the Trotter-Suzuki decomposition^37^ or its variations. We have developed this technique to show that there is at least one efficient method to implement our algorithm. It remains, as future work, to identify optimal implementation methods that may preserve the continuous nature of the algorithm^38^.

$H_A$ can be decomposed as a sum of Hermitian matrices acting on small subspaces

$$\begin{aligned} H_A(F,{|{\psi _j}\rangle },j)=(\gamma +\delta j)\left( \sum _{i=1}^m NS(C_i)-\alpha {|{\psi _0}\rangle }{\langle {\psi _0}|}\right) +A \end{aligned}$$

where $NS(C_i)$ are the Hermitian matrices that compose *M*(*F*). *M*(*F*) and *A*(*n*) (Eq. (1)) seem to be expensive to construct, but in reality *M*(*F*) can be constructed using *O*(*m*) unitary matrices, while *A*(*n*) can be constructed in *O*(*n*). Each $NS(C_i)$ can be built from $2^K$ unitary matrices *acting on a small subspace*, this is because *K* is the number of variables contained in each and every clause of a *K*-SAT instance and, consequently, $2^K$ is a rather small number, certainly much less than $2^n$, where *n* is the number of binary variables over which a *K*-SAT instance is defined. So, for any instance of the *K*-SAT problem *K* is a fixed integer number equal to the number of variables in each and every clause, then $2^K = O(1)$ for any given *K*-SAT instance.

For example, the first clause of $F_1({\mathbf {x}})=(x_1 \vee x_2) \wedge (x_1 \vee \overline{x}_2) \wedge (\overline{x}_1 \vee x_2) \wedge (\overline{x}_2 \vee x_3)$ reads $C_1=(x_1 \vee x_2)$ and the states that represent the assignments that do not satisfy $C_1$ are ${|{000}\rangle }$ and ${|{001}\rangle }$. The sum of their outer products is the matrix $NS(C_1)$

9 $$\begin{aligned} NS(C_1)= & {} {|{0}\rangle }{\langle {0}|}\otimes {|{0}\rangle }{\langle {0}|}\otimes I\nonumber \\= & {} \frac{1}{2}(I+\hat{\sigma }_z)\otimes \frac{1}{2}(I+\hat{\sigma }_z)\otimes I \end{aligned}$$

10 $$\begin{aligned}= & {} \frac{1}{4}(I\otimes I\otimes I+I\otimes \hat{\sigma }_z\otimes I+\hat{\sigma }_z \otimes I\otimes I+ \hat{\sigma }_z\otimes \hat{\sigma }_z\otimes I) \end{aligned}$$

where $\hat{\sigma }_z$ is the Pauli operator $\hat{\sigma }_z = |0\rangle \langle 0 | - |1\rangle \langle 1 |$. So, *M*(*F*) can be expressed as the sum of $2^Km$ Hermitian operators. In general, the number of *I*s in $NS(C_i)$ is $n-K$, in our example (Eq. (9)) $K=2$ and $n=3$ so it has one *I*. For instances with more variables, the number of *I* increases which means that each $NS(C_i)$ only acts in a small subspace. Expressing $NS(C_i)$ as the sum of $2^K$ Hermitian operators, as in Eq. (10), requires using $K2^{K-1}$ Pauli operators $\hat{\sigma }_z$. The number of $\hat{\sigma }_z$ operators in $NS(C_i)$ is independent from the number of variables in the instance, if the instance had more variables it would just add more *I*s to each $NS(C_i)$. Consequently, we do not need an exponential number of Pauli matrices to construct *M*(*F*).

The unitary $e^{i\alpha {|{\psi _j}\rangle }{\langle {\psi _j}|}t}$ can be efficiently implemented using ancilla qubits. A controlled gate applies a NOT gate to an ancilla qubit initially in ${|{0}\rangle }$ if the original *n* qubits are in ${|{\psi _j}\rangle }$ (this can be done with a polynomial number of gates and ancillas^39^). Then, if the ancilla qubit is in ${|{1}\rangle }$ a controlled gate applies $e^{i\alpha t}$ to any of the original *n* qubits. The required projector for lowering the energy of ${|{\psi _j}\rangle }$ can also be built with a gadget proposed by Dodds et al^40^.

## Analytical approximation of the algorithm behavior

To analytically approximate the probability $P_s(t)$ of finding a satisfying assignment to a *K*-SAT instance *F*, which is equivalent to having ${|{s}\rangle }$ as post-measurement quantum state, and to understand how $\gamma +\delta j$ affects this probability, we use the effective Hamiltonian $H_{\text {eff}}$ obtained by degenerate perturbation theory. The effective Hamiltonian only acts on a reduced space and only describes part of the energy spectrum of the complete Hamiltonian. It has the advantage of being simpler than the complete Hamiltonian and it is more general than normal perturbation theory since it takes into account the effects of the off-diagonal elements that link the states of interest. For example, it is used in the Born-Oppenheimer approximation to separate the electronic wavefunction from the nuclear wavefunction of a molecule^41^. In our case we want to separate the subspace spanned by ${|{\psi _j}\rangle }$ and ${|{s}\rangle }$ in $H_A$ to later approximate $P_s(t)$.

Perturbation theory is a set of approximations used to describe a complex quantum system in terms of a simpler one. The approximation is based on the idea of using a Hamiltonian (the unperturbed Hamiltonian $H_0$) with known solutions and adding a perturbation Hamiltonian $H_1$ that slightly modifies the system. The solutions of the perturbed Hamiltonian $H_p$ can be expressed as corrections to the solutions of the unperturbed Hamiltonian $H_0$. Degenerate perturbation theory is a variation of perturbation theory used for unperturbed Hamiltonians with degeneracy in their energy spectrum.

The perturbed Hamiltonian is $H_p=H_0+\lambda H_1$ where $\lambda$ is a dimensionless parameter small enough so that the spectrum of $H_0$ constitutes a good starting point to approximate the spectrum of $H_p$. In the following examples $\lambda$ is set to 1 (as it is frequently done in the literature) in the understanding that the elements of $H_1$ are smaller than those of $H_0$.

As for our algorithm, to approximate the Hamiltonian already presented in Eq. (3)

$$\begin{aligned} H_A=A(n)+(\gamma +\delta j)(M-\alpha {|{\psi _j}\rangle }{\langle {\psi _j}|}) \end{aligned}$$

we use the potential part

$$\begin{aligned} V=(\gamma +\delta j)(M-\alpha {|{\psi _j}\rangle }{\langle {\psi _j}|}) \end{aligned}$$

as the unperturbed Hamiltonian $H_0=V$, and $\lambda H_1=A(n)$ is used as the perturbation Hamiltonian. Hence,

$$\begin{aligned} H_A=H_0+\lambda H_1 \end{aligned}$$

We have chosen $H_0$ and $\lambda H_1$ to be *V* and *A*(*n*) respectively because the entries of $\lambda H_1=A$ are much smaller than those of $H_0=V$. Entries of $H_1=A$ are composed by 1s and 0s while entries of $H_0=V$ have average value potential value calculated as follows:

For big instances, the contribution of $-\alpha {|{\psi _j}\rangle }{\langle {\psi _j}|}$ can be ignored since it only affects one of the $2^n$ possible states. So,

$$\begin{aligned} V\approx M(F). \end{aligned}$$

Also, from Eq. (6), we know that

$$\begin{aligned} M(F)=\sum _{i=1}^{m}NS(C_i). \end{aligned}$$

So, the average potential value is the trace of *M*(*F*) divided by $2^n$.

$$\begin{aligned} apv=\frac{(\gamma +\delta j)\text{tr}(M(F))}{2^n} \end{aligned}$$

To compute $\text{tr}(M(F))$ we need to know how many elements in *M*(*F*) each of the *m* $NS(C_i)$ matrices contribute ($C_i$ are the clauses in *F*). As it can be seen in “Implementation details” Eq. (5), the trace of each $NS(C_i)$ is $\text{tr}(NS(C_i))=2^{n-K}$, where *K* is the number of literals per clause, so

11 $$\begin{aligned} \text{tr}(M(F))= & {} \sum _{i=1}^{m}\text{tr}(NS(C_i))=\sum _{i=1}^{m}2^{n-K}=m2^{n-K}\nonumber \\ apv= & {} \frac{(\gamma +\delta j)m2^{n-K}}{2^n}=\frac{(\gamma +\delta j)m}{2^K}. \end{aligned}$$

which means that the larger the number of variables and clauses, and the higher value of $\gamma +\delta j$, the better the approximation.

It is convenient to use $H_0=V$ since it is a diagonal matrix and calculating its energies and eigenstates is straightforward. Also, there is degeneracy in the energy spectrum of *V* since the energies of ${|{\psi _j}\rangle }$ and ${|{s}\rangle }$ (both eigenstates of $H_0=V$) are $E_s=E_{\psi _j}=0$.

### Effective Hamiltonian via degenerate perturbation theory.

The effective Hamiltonian $H_{\text {eff}}$ acts on a reduced space and only describes part of the energy spectrum of the complete Hamiltonian, which in our case is $H_A$. The effective Hamiltonian we want to approximate is the one spanned by the states ${|{\psi _j}\rangle }$ and ${|{s}\rangle }$ since $H_A$ has two eigenvectors that can be approximated as a linear combination of ${|{\psi _j}\rangle }$ and ${|{s}\rangle }$ by the use of the effective Hamiltonian $H_{\text {eff}}$. The approximated eigenvectors and energies then will be used to approximate $P_s(t)$, the probability of getting ${|{s}\rangle }$ as post-measurement state, i.e., finding a satisfying assignment for a *K*-SAT instance *F*. As before, our analysis is focused on instances with a unique satisfying solution to make it clearer and because those instances are the hardest to solve.

States ${|{\psi _j}\rangle }$ and ${|{s}\rangle }$ may be far from each other in terms of the Hamming distance. If the Hamming distance between ${|{\psi _j}\rangle }$ and ${|{s}\rangle }$ is *d*, i.e. $d_H({|{\psi _j}\rangle },{|{s}\rangle }=d$, then a *d*th order approximation to $H_{\text {eff}}$ is needed for the effects of quantum tunneling between ${|{\psi _j}\rangle }$ and ${|{s}\rangle }$ to be present in $H_{\text {eff}}$. In this analysis we use a 3rd order approximation but the process for higher orders can be easily generalized.

From Gottfried^42^, the spectrum of $H_0=V$ has degeneracy from the energies $E_s=E_{\psi _j}=0$ corresponding to the states ${|{\psi _j}\rangle }$ and ${|{s}\rangle }$. We will call *D* the degenerate subspace in $H_0=V$ spanned by the states $\{{|{\psi _j}\rangle },{|{s}\rangle }\}\in D$.

The perturbed states ${|{a}\rangle }$ that correspond to the degenerate states ${|{\alpha }\rangle }$ (where ${|{\alpha }\rangle }$ represent any of the states in *D*) that split due to the perturbation can be written as the following linear combination

$$\begin{aligned} {|{a}\rangle }=\sum _\alpha c_\alpha {|{\alpha }\rangle }+\sum _\mu d_\mu {|{\mu }\rangle } \end{aligned}$$

where ${|{\mu }\rangle }$ are the states outside *D*.

To obtain the effective Hamiltonian we will approximate $d_\mu$ and do some manipulations so we end up with an eigenvalue problem in the subspace *D*. As we will see, the order of coefficients $c_\alpha$ are *O*(1) while coefficients $d_\mu$ are $O(\lambda )$. Based on this, we will approximate the states ${|{a}\rangle }$ as a linear combination of the degenerate states ${|{\alpha }\rangle }$.

Using $$H_0{|{\alpha }\rangle }=E_\alpha {|{\alpha }\rangle }=0$$ and $$(H_A-E_a){|{a}\rangle }=0$$

12 $$\begin{aligned} (H_A-E_a){|{a}\rangle }=\sum _\alpha c_\alpha (\lambda H_1-E_a) {|{\alpha }\rangle }+\sum _\mu d_\mu (E_\mu -E_a+\lambda H_1){|{\mu }\rangle }=0 \end{aligned}$$

Projecting (12) onto a state ${|{\beta }\rangle }$ in *D*, and a state ${|{\nu }\rangle }$ outside *D* gives the equations

13 $$\begin{aligned}&-c_\beta E_a+\lambda \sum _\alpha c_\alpha {\langle {\beta }|}H_1{|{\alpha }\rangle }+\lambda \sum _\mu d_\mu {\langle {\beta }|}H_1{|{\mu }\rangle }=0 \end{aligned}$$

14 $$\begin{aligned}&d_\nu (E_\nu -E_a)+\lambda \sum _\alpha c_\alpha {\langle {\nu }|}H_1{|{\alpha }\rangle }+\lambda \sum _\mu d_\mu {\langle {\nu }|}H_1{|{\mu }\rangle }=0 \end{aligned}$$

Since $d_\mu =O(\lambda )$ the last term in (14) is $O(\lambda ^2)$ and can be drooped for the approximation. Since the difference between $E_\alpha$ and $E_a$ is negligible compared to $E_\nu -E_a$, $E_a$ can be replaced by $E_\alpha =0$

15 $$\begin{aligned} d_\nu =-\lambda \sum _\alpha \frac{c_\alpha {\langle {\nu }|}H_1{|{\alpha }\rangle }}{E_\nu } \end{aligned}$$

Substituting (15) in (14) we obtain a better approximation of $d_\nu$ than (15). We can iteratively repeat this process to generate higher order approximations of $d_\nu$ (and in turn, as we will see in Eq. (18), higher order approximations of $H_{\text {eff}}$).

16 $$\begin{aligned}&d_\nu E_\nu +\lambda \sum _\alpha c_\alpha {\langle {\nu }|}H_1{|{\alpha }\rangle }-\lambda ^2\sum _\alpha c_\alpha \sum _\mu \frac{ {\langle {\nu }|}H_1{|{\mu }\rangle } {\langle {\mu }|}H_1{|{\alpha }\rangle }}{E_\mu }=0\nonumber \\&\quad d_\nu =-\lambda \sum _\alpha c_\alpha \frac{{\langle {\nu }|}H_1{|{\alpha }\rangle }}{E_\nu }+\lambda ^2\sum _\alpha c_\alpha \sum _\mu \frac{ {\langle {\nu }|}H_1{|{\mu }\rangle } {\langle {\mu }|}H_1{|{\alpha }\rangle }}{E_\mu E_\nu } \end{aligned}$$

Noting $d_\nu$ and $d_\mu$ both represent the coefficients of the states outside *D*, we can substitute (16) in (13)

17 $$\begin{aligned} -c_\beta E_a+\sum _\alpha c_\alpha \Biggr ( \lambda {\langle {\beta }|}H_1{|{\alpha }\rangle }-\lambda ^2\sum _\mu \frac{ {\langle {\beta }|}H_1{|{\mu }\rangle } {\langle {\mu }|}H_1{|{\alpha }\rangle }}{E_\mu } +\lambda ^3\sum _\mu \sum _\nu \frac{ {\langle {\beta }|}H_1{|{\mu }\rangle } {\langle {\mu }|}H_1{|{\nu }\rangle }{\langle {\nu }|}H_1{|{\alpha }\rangle }}{E_\mu E_\nu } \Biggr )=0 \end{aligned}$$

This is equivalent to the eigenvalue problem (13)

$$\begin{aligned} -c_\beta E_a+\lambda \sum _\alpha c_\alpha {\langle {\beta }|}H_1{|{\alpha }\rangle }+\lambda \sum _\mu d_\mu {\langle {\beta }|}H_1{|{\mu }\rangle }=0 \end{aligned}$$

For the third order approximation of the effective Hamiltonian $H_{\text {eff}}^{(3)}$ in the subspace *D* ( which is 2 dimensional in this case since we have a 2-fold degeneracy), that is, a Hamiltonian that only acts in this reduced space and only describes part of the spectrum of the true Hamiltonian $H_A$.

18 $$\begin{aligned} {\langle {\beta }|}H_{\text {eff}}^{(3)}{|{\alpha }\rangle }=\lambda {\langle {\beta }|}H_1{|{\alpha }\rangle }-\lambda ^2\sum _\mu \frac{ {\langle {\beta }|}H_1{|{\mu }\rangle } {\langle {\mu }|}H_1{|{\alpha }\rangle }}{E_\mu }+\lambda ^3\sum _\mu \sum _\nu \frac{ {\langle {\beta }|}H_1{|{\mu }\rangle } {\langle {\mu }|}H_1{|{\nu }\rangle }{\langle {\nu }|}H_1{|{\alpha }\rangle }}{E_\mu E_\nu } \end{aligned}$$

For a *d*th order approximation, when the distance between the two states in *D*, ${|{\psi _j}\rangle }$ and ${|{s}\rangle }$, is $d_H=d$, the off diagonal elements of $H_{\text {eff}}^{(d)}$ in the subspace D will only have non zero contributions when the states evaluated in the *dth* order term are those states that form a tunneling path of length *d* from ${|{\psi _j}\rangle }$ to ${|{s}\rangle }$. Without the off-diagonal elements in $H_{\text {eff}}^{(d)}$ the approximation would not include the effects of tunneling from ${|{\psi _j}\rangle }$ to ${|{s}\rangle }$.

### Approximation of the probability of finding a satisfying assignment to a *K*-SAT instance

We now present an approximation of the probability $P_s(t)$, the probability of finding a satisfying assignment to a *K*-SAT instance *F*, which is equivalent to obtain ${|{s}\rangle }$ as post-measurement state of a quantum evolution process starting with the quantum state ${|{\psi _j}\rangle }$. For this purpose, we use the effective Hamiltonian $H_{\text {eff}}$ from Eq. (18) for three different cases: $d_H({|{\psi _j}\rangle },{|{s}\rangle })=3$, $d_H({|{\psi _j}\rangle },{|{s}\rangle })=2$, and $d_H({|{\psi _j}\rangle },{|{s}\rangle })=1$. The approximation for $P_s(t)$ for distances not presented here can be easily obtained by slightly modifying the procedure.

First we show the approximation $H_{\text {eff}}^{(3)}$ for $d_H({|{\psi _j}\rangle },{|{s}\rangle })=3$. As stated in the beginning of this section, $H_0=V=(\gamma +\delta j)(M-\alpha {|{\psi _j}\rangle }{\langle {\psi _j}|})$, and $\lambda H_1=A(n)$.

19 $$\begin{aligned} H_{\text {eff}}^{(3)}=A-\sum _\mu \frac{ A{|{\mu }\rangle } {\langle {\mu }|}A}{E_\mu }+\sum _\mu \sum _\nu \frac{ A{|{\mu }\rangle } {\langle {\mu }|}A{|{\nu }\rangle }{\langle {\nu }|}A}{E_\mu E_\nu } \end{aligned}$$

where ${|{\mu }\rangle }$ and ${|{\nu }\rangle }$ are eigenvectors with eigenvalues $E_\mu ,E_\nu$ in $H_0=V$ different from ${|{\psi _j}\rangle }$ and ${|{s}\rangle }$. The resulting effective Hamiltonian will be $2\times 2$ since it is from the subspace spanned by $\{{|{\psi _j}\rangle },{|{s}\rangle }\}$ from which we will approximate two eigenvectors of $H_A$ as a linear superposition of ${|{\psi _j}\rangle }$ and ${|{s}\rangle }$, and later use them to approximate $P_S(t)$.

The linear term in (19) has no contribution to the diagonal of $H_{\text {eff}}$ since *A* (as any adjacency matrix) has a diagonal full of 0s. As for the off diagonal elements of the linear term, there is no way for ${|{\psi _j}\rangle }$ and ${|{s}\rangle }$ to tunnel from one to the other without going through intermediate states in the case $d_H=3$, so there are no off diagonal elements contributed by the lineal term.

Evaluating the second order term, we can see it only has diagonal elements, the contributions to the diagonal come from evaluating the term with states at $d_H({|{\mu }\rangle }, {|{s}\rangle })=1$ or $d_H({|{\mu }\rangle },{|{\psi _j}\rangle })=1$. Each contribution to this term is divided by its corresponding $E_\mu$ which is a product of $\gamma +\delta j$ and the number of clauses the corresponding state ${|{\mu }\rangle }$ does not satisfy.

The third order term is the only contributor to the off-diagonal elements of $H_{\text {eff}}$ in the case $d_H=3$. These contributions come from evaluating the third order term with the states that form the shortest tunneling paths from ${|{\psi _j}\rangle }$ to ${|{s}\rangle }$, these states are the ones at a distance $d_H=2$ from ${|{\psi _j}\rangle }$ or ${|{s}\rangle }$ and $d_H=1$ from the other. The easier it is for the states ${|{\psi _j}\rangle }$ and ${|{s}\rangle }$ to tunnel through these paths (when the potential barriers are smaller) the greater the value of the off-diagonal elements will be. We can check the previous statement from Eq. (19) since every contribution from each tunneling path is 1 divided by $E_\mu E_\nu$.

The resulting effective Hamiltonian $H_{\text {eff}}$ in the sub space spanned by $\{{|{\psi _j}\rangle },{|{s}\rangle }\}$ is

20 $$\begin{aligned} H_{\text {eff}}^{(3)}&= \begin{bmatrix} 0 &{} 0\\ 0 &{} 0 \end{bmatrix} + \begin{bmatrix} \frac{v_{11}}{\gamma +\delta j} &{} 0\\ 0 &{} \frac{v_{22}}{\gamma +\delta j} \end{bmatrix} + \begin{bmatrix} 0 &{} \frac{v_{12}}{(\gamma +\delta j)^2}\\ \frac{v_{21}}{(\gamma +\delta j)^2} &{} 0 \end{bmatrix} \nonumber \\ H_{\text {eff}}^{(3)}&= \begin{bmatrix} \frac{v_{11}}{\gamma +\delta j} &{} \frac{v_{12}}{(\gamma +\delta j)^2}\\ \frac{v_{21}}{(\gamma +\delta j)^2} &{} \frac{v_{22}}{\gamma +\delta j} \end{bmatrix} \end{aligned}$$

where $v_{ij}$ are the values of the effective Hamiltonian for $\gamma +\delta j=1$. Since $H_{\text {eff}}$ is Hermitian and the entries are real numbers then $v_{12}=v_{21}$.

Finding the energies of Eq. (20) is straightforward

$$\begin{aligned} \begin{vmatrix} \Updelta v_{11}-E_\pm&\Updelta ^2 v_{12}\\ \Updelta ^2 v_{21}&\Updelta v_{22}-E_\pm \end{vmatrix} = \Updelta ^2 v_{11}v_{22}+E_\pm (-\Updelta v_{11}-\Updelta v_{22})+E_\pm ^2-\Updelta ^4v_{12}^2=0 \end{aligned}$$

21 $$\begin{aligned} E_\pm =\frac{\Updelta }{2}\left( v_{11}+v_{22}\pm \sqrt{(v_{11}-v_{22})^2+4\Updelta ^2v_{12}^2}\right) \end{aligned}$$

where $\Updelta =\frac{1}{\gamma +\delta j}$ for simplicity. To obtain the eigenstates

$$\begin{aligned} \begin{bmatrix} \Updelta v_{11}-E_\pm &{} \Updelta ^2 v_{12}\\ \Updelta ^2 v_{21} &{} \Updelta v_{22}-E_\pm \end{bmatrix} \begin{bmatrix} a\\ b \end{bmatrix} =0 \end{aligned}$$

22 $$\begin{aligned}&(\Updelta v_{11}-E_\pm )a+\Updelta ^2v_{12}b&=0 \end{aligned}$$

23 $$\begin{aligned}&\Updelta ^2 v_{12}a+(\Updelta v_{22}-E_\pm )b&=0 \end{aligned}$$

From Eqs. (22) and (23), eigenvectors are computed

$$ \begin{aligned}    & b(E_{ \pm }  - \Delta v_{{22}} ) = a\Delta v_{{12}}  \\     & b\Delta ^{2} v_{{12}}  = a(E_{ \pm }  - \Delta v_{{11}} ) \\     & |\phi _{ \pm } \rangle  = k_{ \pm } \left[ {\begin{array}{*{20}c}    {E_{ \pm }  - \Delta v_{{22}} }  \\    {\Delta ^{2} v_{{12}} }  \\   \end{array} } \right] = k^{\prime}_{ \pm } \left[ {\begin{array}{*{20}c}    {\Delta ^{2} v_{{12}} }  \\    {E_{ \pm }  - \Delta v_{{11}} }  \\   \end{array} } \right] \\  \end{aligned}  $$

where $k_\pm$ and $k'_\pm$ the are normalization constants

$$ \begin{aligned}   k_{ \pm }  &  = (\Delta ^{4} v_{{12}}^{2}  + (E_{ \pm }  - \Delta v_{{22}} )^{2} )^{{ - \frac{1}{2}}} , \\    k^{\prime}_{ \pm }  &  = (\Delta ^{4} v_{{12}}^{2}  + (E_{ \pm }  - \Delta v_{{11}} )^{2} )^{{ - \frac{1}{2}}}  \\  \end{aligned}  $$

and can be related as follows:

24 $$\begin{aligned} k'_\pm =&(\Updelta ^4v_{12}^2+(E_\pm -\Updelta v_{11})^2)^{-\frac{1}{2}}\nonumber \\ =&\left( \Updelta ^4v_{12}^2+\left( \frac{\Updelta }{2}\left( v_{11}+v_{22}\pm \sqrt{(v_{11}-v_{22})^2+4\Updelta ^2v_{12}^2}\right) -\Updelta v_{11}\right) ^2\right) ^{-\frac{1}{2}}\nonumber \\ =&\left( \Updelta ^4v_{12}^2+\left( \frac{\Updelta }{2}\left( v_{22}-v_{11}\pm \sqrt{(v_{11}-v_{22})^2+4\Updelta ^2v_{12}^2}\right) \right) ^2\right) ^{-\frac{1}{2}}\nonumber \\ =&\left( \Updelta ^4v_{12}^2+(-1)^2\left( \frac{\Updelta }{2}\left( v_{22}+v_{11}\mp \sqrt{(v_{11}-v_{22})^2+4\Updelta ^2v_{12}^2}\right) \right) ^2\right) ^{-\frac{1}{2}}\nonumber \\ =&k_\mp ,~ -k_\mp \end{aligned}$$

The approximated eigenstates of $H_A$, ${|{\phi _\pm }\rangle }$ written as a linear combination of ${|{\psi _j}\rangle }$ and ${|{s}\rangle }$ are

$$\begin{aligned} {|{\phi _\pm }\rangle }=k_\pm (E_\pm -\Updelta v_{22}){|{\psi _j}\rangle }+k_\pm \Updelta ^2v_{12}{|{s}\rangle }=k'_\pm \Updelta ^2v_{12}{|{\psi _j}\rangle }+k_\pm \Updelta ^2v_{12}{|{s}\rangle } \end{aligned}$$

From Eq. (24) and orthogonality, the eigenvectors can be rewritten as

$$\begin{aligned} {|{\phi _+}\rangle }&=k_-\Updelta ^2v_{12}{|{\psi _j}\rangle }+k_+\Updelta ^2v_{12}{|{s}\rangle }\\ {|{\phi _-}\rangle }&=-k_+\Updelta ^2v_{12}{|{\psi _j}\rangle }+k_-\Updelta ^2v_{12}{|{s}\rangle } \end{aligned}$$

The state ${|{\psi _j}\rangle }$ can be written as a linear combination of ${|{\phi _\pm }\rangle }$

$$\begin{aligned}&{{|{\psi _j}\rangle }=\frac{k_-}{gk_+}{|{\phi _+}\rangle }-\frac{1}{g}{|{\phi _-}\rangle }}\\&{g=\Updelta ^2k_+v_{12}\left( 1+\left( \frac{k_-}{k_+}\right) ^2\right) } \end{aligned}$$

where *g* is a normalization constant.

We can use ${|{\phi _\pm }\rangle }$ to approximate the evolution of the real eigenstates of the Hamiltonian $H_A$. The evolution of a true eigenstate of $H_A$ is

$$\begin{aligned} e^{-iH_At}{|{a_\pm }\rangle }=e^{-iE_{a_\pm }t}{|{a_\pm }\rangle } \end{aligned}$$

by substituting the eigenstates and energies by their approximation we obtain

$$\begin{aligned} e^{-iH_At}{|{\phi _\pm }\rangle }\approx e^{-iE_\pm t} {|{\phi _\pm }\rangle } \end{aligned}$$

Using it, we can get an approximation of probability $P_s^{(3)}(t)$ of measuring ${|{s}\rangle }$ at a time *t* evolving from ${|{\psi _j}\rangle }$, when $d_H({|{\psi _j}\rangle },{|{s}\rangle })=3$.

$$ \begin{aligned}    & P_{s}^{{(3)}} (t) = |\langle s|e^{{ - iHt}} |\psi _{j} \rangle |^{2}  \\    P_{s}^{{(3)}} (t) &  \approx \left| {\frac{{k_{ - } }}{{gk_{ + } }}e^{{ - iE_{ + } t}} \langle s||\phi _{ + } \rangle  - \frac{1}{g}e^{{ - iE_{ - } t}} \langle s||\phi _{ - } \rangle } \right|^{2}  \\     &  \approx \left| {\frac{{\Delta ^{2} k_{ - } v_{{12}} }}{g}e^{{ - iE_{ + } t}}  - \frac{{\Delta ^{2} k_{ - } v_{{12}} }}{g}e^{{ - iE_{ - } t}} } \right|^{2}  \\     &  \approx \left| {\frac{{\Delta ^{2} k_{ - } v_{{12}} }}{g}\left( {e^{{ - iE_{ + } t}}  - e^{{ - iE_{ - } t}} } \right)} \right|^{2}  \\     &  \approx \left( {\frac{{\Delta ^{2} k_{ - } v_{{12}} }}{g}} \right)^{2} \left( {2 - 2\cos ((E_{ + }  - E_{ - } )t)} \right) \\  \end{aligned}  $$

That is,

25 $$\begin{aligned} P_s^{(3)}(t)\approx \left( \frac{k_+k_-}{k_-^2+k_+^2}\right) ^2\left( 2-2\cos ((E_+-E_-)t)\right) \end{aligned}$$

The frequency of the probability $P_s^{(3)}(t)$ is approximately

$$\begin{aligned} E_+-E_-=\Updelta \sqrt{(v_{11}-v_{22})^2+4\Updelta ^2v_{12}^2} \end{aligned}$$

Since $\Updelta =\frac{1}{\gamma +\delta j}$, for $\gamma +\delta j>>1\Rightarrow E_+-E_- \approx O(\Updelta )$ since the term with $\Updelta ^2$ vanishes.

Now we briefly show the cases with $d_H=2$ and $d_H=1$ and how their respective equation for $P_s(t)$ compares with $P_s^{(3)}(t)$.

It is easy to follow a similar process to calculate $P_s^{(2)}(t)$, the case with $d_H({|{\psi _j}\rangle },{|{s}\rangle })=2$. In this case at least a second order approximation is needed for the effects of tunneling (the off diagonal elements) to appear in $H_{\text {eff}}^{(2)}$ .

$$\begin{aligned} H_{\text {eff}}^{(2)}=A-\sum _\mu \frac{ A{|{\mu }\rangle } {\langle {\mu }|}A}{E_\mu } \end{aligned}$$

The corresponding $$H_{\text {eff}}$$ is slightly different to the previous case which has an impact in the frequency of $$P_s^{(2)}(t)$$. The first order contribution to $$H_{\text {eff}}$$, as in the previous case, is also null for the same reasons. In this case the second order term gives all the elements for $$H_{\text {eff}}$$. The contributions from the second order come from evaluating the states at $$d_H({|{\mu }\rangle },{|{\psi _j}\rangle })=1$$ or $$d_H({|{\mu }\rangle },{|{s}\rangle })=1$$. Some of these states will be part of the shortest tunneling paths from $${|{\psi _0}\rangle }$$ to $${|{s}\rangle }$$, meaning $$d_H({|{\mu }\rangle },{|{\psi _j}\rangle })=d_H({|{\mu }\rangle },{|{s}\rangle })=1$$, and are the ones that contribute to the off-diagonal elements. As in the previous case ($$d_H=3)$$, the easier it is for $${|{\psi _j}\rangle }$$ to tunnel through a barrier to get to $${|{s}\rangle }$$ the greater the contribution from that path. Each contribution is divided by the corresponding $$E_\mu $$, that is a product of $$\gamma +\delta j$$ and the number of clauses the corresponding state $${|{\mu }\rangle }$$ does not satisfy. This gives the following effective Hamiltonian

26 $$\begin{aligned} H_{\text {eff}}^{(2)}= \begin{bmatrix} \frac{v_{11}}{\gamma +\delta j} &{} \frac{v_{12}}{\gamma +\delta j}\\ \frac{v_{21}}{\gamma +\delta j} &{} \frac{v_{22}}{\gamma +\delta j} \end{bmatrix} \end{aligned}$$

Following a process similar to the one presented for $d_H=3$, we obtain

$$\begin{aligned} P_s^{(2)}(t)\approx \left( \frac{k_+k_-}{k_-^2+k_+^2}\right) ^2\left( 2-2\cos ((E_+-E_-)t)\right) \end{aligned}$$

where

$$\begin{aligned}&E_\pm =\frac{\Updelta }{2}\left( v_{11}+v_{22}\pm \sqrt{(v_{11}-v_{22})^2+4v_{12}^2}\right) \\&k_\pm =(\Updelta ^2v_{12}^2+(E_\pm -\Updelta v_{22})^2)^{-\frac{1}{2}} \end{aligned}$$

$P_s^{(2)}(t)$ has a frequency $E_+-E_-=\Updelta \sqrt{(v_{11}-v_{22})^2+4v_{12}^2}$ which is $O(\Updelta )$, Comparing it to the $P_s^{(3)}(t)$ frequency, when $\gamma +\delta j>>1$, remembering $\Updelta =\frac{1}{\gamma +\delta j}$, $P_{s}^{(2)}(t)$ has the term $4v_{12}^2$ inside the square root compared to $P_{s}^{(3)}(t)$ where $\Updelta ^24v_{12}^2$ vanishes. Also, the off-diagonal elements $v_{12}$ from $H_{\text {eff}}$ when $d_H=2$ tend to be greater compared to the case $d_H=3$ since there are less potential barriers in the tunneling path from ${|{\psi _j}\rangle }$ to ${|{s}\rangle }$. Consequently, in most cases the frequency of $P_{s}^{(2)}(t)$ will be higher than he frequency of $P_{s}^{(3)}(t)$.

For $d_H=1$, the first order approximation can be used. The $H_{\text {eff}}$ is simply

$$\begin{aligned} H_{\text {eff}}^{(1)}= \begin{bmatrix} 0 &{} 1\\ 1 &{} 0 \end{bmatrix} \end{aligned}$$

The probability $P_s^{(1)}(t)$ can be obtained as follows

27 $$\begin{aligned} P_s^{(1)}(t)&\approx |{\langle {s}|}e^{-i\sigma _x t} {|{\psi _j}\rangle }|^2 \nonumber \\&\approx {\langle {s}|}e^{-i\sigma _x t} {|{\psi _j}\rangle } {\langle {\psi _j}|} e^{i\sigma _x t} {|{s}\rangle }\nonumber \\&\approx {\langle {s}|} (\cos (t) {|{\psi _j}\rangle } -i \sin (t) {|{s}\rangle }) (\cos (t){\langle {\psi _j}|}+i\sin (t) {\langle {s}|}){|{s}\rangle }\nonumber \\ P_s^{(1)}(t)&\approx \sin ^2(t) \end{aligned}$$

Equation (27) does not have dependency on $\Updelta$ and has a period of $\pi$. Hence, for $t=\frac{\pi }{2}+\pi m$, where $m \in {\mathbb {Z}}$, $P_s^{(1)}(t)$ is at its highest value. As $\gamma +\delta j$ increases, the potential barriers around the states ${|{\psi _j}\rangle }$ and ${|{s}\rangle }$ get larger, reducing the tunneling to the states outside of *D* (for $d_H=1$ the states in *D* are neighbors) which makes the real behavior of $P_s^{(1)}(t)$ closer to the approximation of Eq. (27). Due to this lack of dependency on $\Updelta$ from the approximation of $P_s^{(1)}(t)$, the actual period of $P_s^{(1)}(t)$ (without approximations) is practically always the same. With this in mind, the values of $\gamma$ and $\delta$ have to keep the evolution time $t_f=\frac{\pi }{2}+\pi m$, where $m \in {\mathbb {Z}}$, as a time that greatly amplifies $P_s(t)$ for multiple values of $d_H({|{\psi _j}\rangle },{|{s}\rangle })$. In fact, our numerical simulations using the optimal parameters, found by doing a sweep, show this property of preserving $t_f$ as an evolution time that greatly amplifies $P_s(t)$. Our simulations give us an optimal measurement frequency of $t_f=4.72$, that is approximately $\frac{3}{2}\pi$, in agreement with analytical results. Keep in mind that for larger numbers of variables and clauses this value may change to $t_f=\frac{\pi }{2}+\pi m$, with a different value of *m*.

### Comparisons between the analytical approximation and simulations

In this section, we compare the analytical approximations of $P_s(t)$ from the previous section

$$ \begin{aligned}   P_{s}^{{(3)}} (t) &  \approx \left( {\frac{{k_{ + } k_{ - } }}{{k_{ - }^{2}  + k_{ + }^{2} }}} \right)^{2} \left( {2 - 2\cos (t\Delta \sqrt {(v_{{11}}  - v_{{22}} )^{2}  + 4\Delta ^{2} v_{{12}}^{2} } )} \right) \\    P_{s}^{{(2)}} (t) &  \approx \left( {\frac{{k_{ + } k_{ - } }}{{k_{ - }^{2}  + k_{ + }^{2} }}} \right)^{2} \left( {2 - 2\cos (t\Delta \sqrt {(v_{{11}}  - v_{{22}} )^{2}  + 4v_{{12}}^{2} } )} \right) \\    P_{s}^{{(1)}} (t) &  \approx \sin ^{2} (t) \\  \end{aligned}  $$

to our numerical simulations of $P_s(t)$ for the distances $d_H({|{\psi _j}\rangle },{|{s}\rangle })=1,2,3$. The instance used for these comparisons is the following 6 variable instance with 24 clauses and the unique satisfying assignment $s=[110111]$.

28 $$\begin{aligned} F_3({\mathbf {x}})= & {} (\overline{x}_2 \vee \overline{x}_3 \vee \overline{x}_4) \wedge (\overline{x}_1 \vee \overline{x}_3 \vee x_6) \wedge (\overline{x}_2 \vee x_5 \vee \overline{x}_6) \wedge (x_1 \vee \overline{x}_2 \vee \overline{x}_4) \wedge (x_2 \vee \overline{x}_5 \vee x_6) \wedge \nonumber \\&(x_3 \vee x_5 \vee x_6) \wedge (\overline{x}_1 \vee x_4 \vee x_5) \wedge (x_1 \vee \overline{x}_4 \vee x_5) \wedge (x_3 \vee \overline{x}_4 \vee x_6) \wedge (\overline{x}_1 \vee \overline{x}_5 \vee x_6) \wedge \nonumber \\&(x_2 \vee \overline{x}_4 \vee \overline{x}_5) \wedge (\overline{x}_4 \vee x_5 \vee \overline{x}_6) \wedge (\overline{x}_1 \vee x_5 \vee x_6) \wedge (x_1 \vee x_3 \vee x_5) \wedge (\overline{x}_1 \vee x_4 \vee x_6) \wedge \nonumber \\&(\overline{x}_2 \vee x_3 \vee x_4) \wedge (\overline{x}_1 \vee x_2 \vee \overline{x}_6) \wedge (\overline{x}_3 \vee x_4 \vee \overline{x}_5) \wedge (x_2 \vee x_3 \vee x_4) \wedge (\overline{x}_1 \vee \overline{x}_3 \vee \overline{x}_6) \wedge \nonumber \\&(\overline{x}_2 \vee x_3 \vee x_5) \wedge (\overline{x}_1 \vee x_2 \vee \overline{x}_6) \wedge (\overline{x}_1 \vee x_2 \vee x_4) \wedge (x_1 \vee x_4 \vee x_5) \end{aligned}$$

To exemplify the case $d_H=3$ we used the state ${|{\psi _j}\rangle }={|{010001}\rangle }$. The effective Hamiltonian using degenerate perturbation theory up to the third order, as in Eq. (20), is

$$\begin{aligned} H_{\text {eff}}^{(3)}= \begin{bmatrix} \frac{-2.455}{\gamma +\delta j} &{} \frac{2.555}{(\gamma +\delta j)^2}\\ \frac{2.555}{(\gamma +\delta j)^2} &{} \frac{-3.666}{\gamma +\delta j} \end{bmatrix} \end{aligned}$$

**Figure 1:**
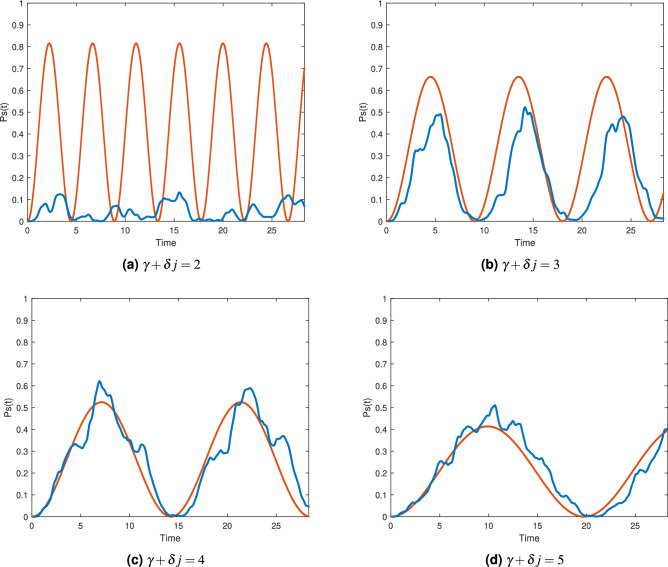
Comparison between simulations (blue line) and the analytical approximation (orange line) of $P_s(t)$ for initial state ${|{010001}\rangle }$, $d_H=3$.

Figure 1 compares analytical approximation and numerical simulations of $P_s(t)$ evolving from ${|{\psi _j}\rangle }={|{010001}\rangle }$ for various values of $\gamma +\delta j$. As the value of $\gamma +\delta j$ increases the approximation gets better since the tunneling to states outside the main tunneling paths from ${|{\psi _j}\rangle }$ to ${|{s}\rangle }$ decreases, and the reflections at the potential barriers also decrease. This contributes to reduce the small oscillations seen in the numerical simulations of $P_s(t)$. We can also see that even for small values of $\gamma +\delta j$, the approximation of the period is accurate.

For $d_H=2$, we use the initial state ${|{\psi _j}\rangle }={|{110001}\rangle }$. By using perturbation theory up to the second order, as in Eq. (26), we obtain the following effective Hamiltonian

$$\begin{aligned} H_{\text {eff}}^{(2)}= \begin{bmatrix} \frac{-0.446}{\gamma +\delta j} &{} \frac{-0.266}{\gamma +\delta j}\\ \frac{-0.266}{\gamma +\delta j} &{} \frac{-0.733}{\gamma +\delta j} \end{bmatrix} \end{aligned}$$

**Figure 2:**
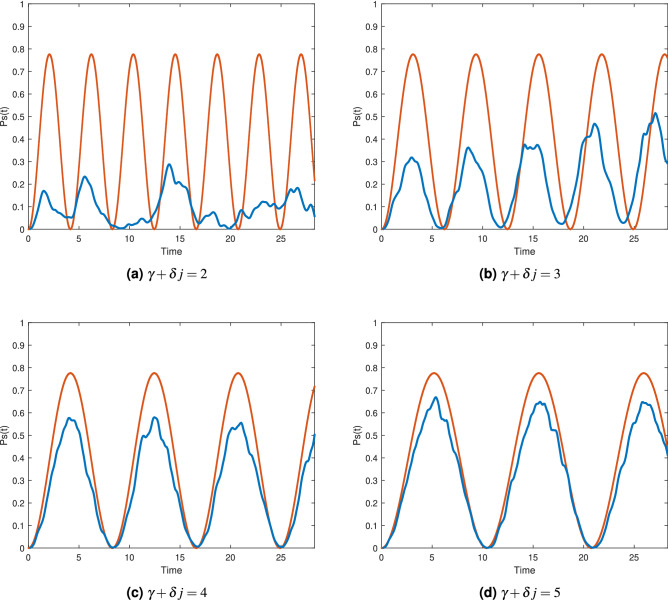
Comparison between simulations (blue line) and the analytical approximation (orange) of $P_s(t)$ for initial state ${|{110001}\rangle }$, $d_H=2$.

Figure 2 shows comparisons between the analytical approximation and the numerical simulations of $P_s(t)$ evolving from ${|{110001}\rangle }$ for various values of $\gamma +\delta j$.

Lastly, for $d_H=1$ and using the initial state ${|{110011}\rangle }$ the effective matrix is simply

$$\begin{aligned}H_{\text {eff}}^{(1)}= \begin{bmatrix} 0 &{} 1\\ 1 &{} 0 \end{bmatrix} \end{aligned}$$

**Figure 3:**
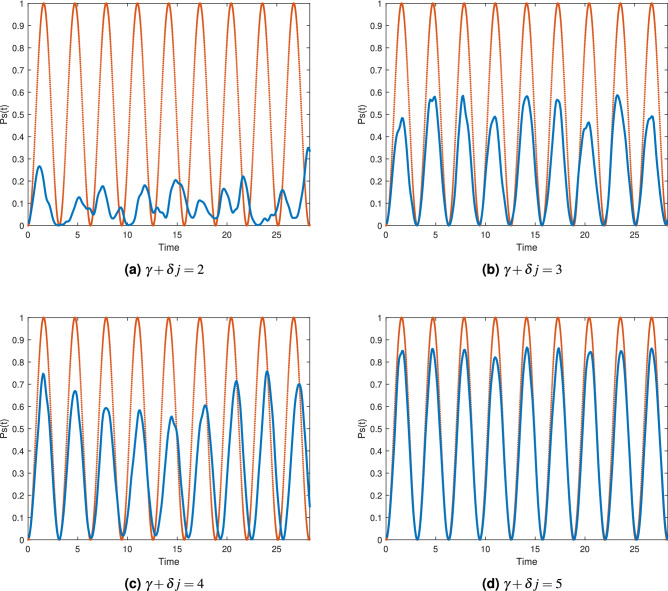
Comparison between simulations (blue line) and the analytical approximation (orange) of $P_s(t)$ for a Hamming distance of 1.

Figure 3 shows comparisons between the analytical approximation and the numerical simulations of $P_s(t)$ evolving from ${|{110011}\rangle }$ for various values of $\gamma +\delta j$

Graphs from Figs. (1,2,3) show the advantage of including potential barriers compared to the exponentially small overlap $P_s(t)=\sin ^6(t) \cos ^6(t) = \sin ^6(2t)/2^6$, obtained from Eq. (2), corresponding to solely using the hypercube adjacency matrix as the quantum walk Hamiltonian.

For distances $d_H>3$, the patterns exhibited in Figs. 1, 2 and 3 hold. In general, the period of $P_s(t)$ grows longer as $d_H$ increases as a consequence of having to tunnel through longer paths, which is also reflected in the analytical approximation for the period by becoming more dependent on $\gamma +\delta j$ as a consequence of $H_{\text {eff}}$ having the mathematical structure

29 $$\begin{aligned} H_{\text {eff}}^{(d)}= \begin{bmatrix} \frac{v_{11}}{\gamma +\delta j} &{} \frac{v_{12}}{(\gamma +\delta j)^{d-1}}\\ \frac{v_{21}}{(\gamma +\delta j)^{d-1}} &{} \frac{V_{22}}{\gamma +\delta j} \end{bmatrix}, \end{aligned}$$

where *d* is the distance $d_H({|{\psi _j}\rangle },{|{s}\rangle })=d$ and also the minimum approximation order necessary for the tunneling effects to be present in the approximation. As $d_H=d$ grows the off-diagonal elements in $H_{{\text {eff}}}$ that serve as couplings between ${|{\psi _j}\rangle }$ and ${|{s}\rangle }$ get smaller which implies longer periods for transitioning between states.

## Numerical calculation of parameters and additional simulations

So far, we have been able to numerically estimate optimal values for parameters $\gamma$ and $\delta$; moreover, our analytical approximation of $t_f=\frac{\pi }{2}+\pi m$, $m \in {\mathbb {Z}}$ has multiple possible values. Since it remains as future work to derive analytical formulae for these parameters, we have approximated three sets of these parameters for 3-SAT instances of 4, 6, and 10 variables, with 16, 24, and 40 clauses respectively.

Our database, fully available in^32^, consists of thousands of 3-SAT instances from which 5799 have 4 variables and 16 clauses, 1949 have 6 variables and 24 clauses, and 606 have 10 variables and 40 clauses. Every instance in the database has a unique satisfying solution. All instances were randomly created by a uniform distribution, and then solved by exhaustive substitution as our instances are small enough for that. Instances with unique satisfying assignment were saved for the database.

The sets of parameters were computed by doing thousands of simulations on 3-SAT instances for the three different number of variables in our data set. The parameters were computed by slightly varying the value of each parameter and running 500 simulations for each combination of parameters. Half the dataset was used to find the parameters while the other half was used to test their performance. Each simulation was run using a randomly selected instance from the dataset, starting with a randomly selected initial state, and stopped after *n* measurements, where *n* is the number of variables of the instance, meaning only the first run of the algorithm (without resetting after *n* iterations) was considered for these calculations.

In every case the measurement frequency $t_f$ was around 4.72 which is approximately $\frac{3}{2}\pi$ in accordance to our analytical approximation. Future versions of the algorithm may make use of variable measurement times, possibly by the use of techniques used in the literature of first detection time for quantum walks^43,44^.

The optimal sets of parameters we estimated for each number of variables and clauses are shown in Table 2. Alongside them we have included statistical information from 1000 simulations for each of the three different number of variables: The success rate of having ${|{s}\rangle }$ as post-measurement quantum state (that is, the success rate of finding the unique solution of each instance used in this study) for the first run of the algorithm (*n* iterations at most), average number of necessary iterations to get ${|{s}\rangle }$ as post-measurement quantum state, and the standard deviation in the number of iterations to get ${|{s}\rangle }$ as post-measurement quantum state.

**Table 2:** Optimal parameters and statistical information from 1000 simulations.

*n*	Optimal $\gamma$	Optimal $\delta$	Success rate	Avg. no. of iterations	Std in no. of iterations
4	2.6	0.5	0.87	2.43	0.76
6	1	0.6	0.71	4.04	1.29
10	1	0.45	0.42	5.88	1.88

**Figure 4:**
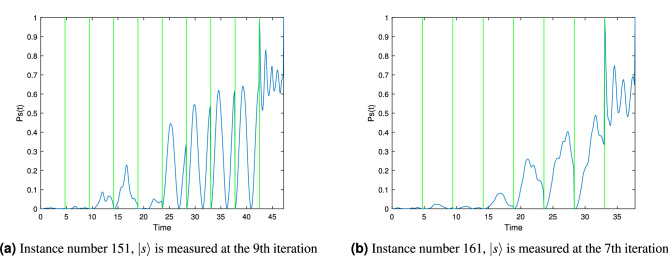
Probabilities of measuring ${|{s}\rangle }$ depicting the behavior of the complete algorithm for 2 randomly selected 10 variables 3-SAT instances with a unique solution from our database. Vertical green lines show when a measurement took place.

Taking in account all the simulations we ran for Table 2, the success rate was 0.66 for the first run of the algorithm. The success rate for the 10 variable instances may seem low, but remember it is only for the first run, for the second run its success rate increases to 0.63 and the fifth run is already 0.93. Also, we are using linear increments for $\gamma +\delta j$, the use of a different function may translate into a better performance. Figure 4 presents simulations of $P_s(t)$ for two randomly selected 10 variables 3-SAT instances from the database^32^ showing the expected behavior for runs where the satisfactory state ${|{s}\rangle }$ is measured.

## Conclusions

We have presented a quantum walk-based algorithm that explicitly utilizes potential barriers and quantum tunneling to find satisfying assignments for hard *K*-SAT instances.

One of the novelties of our paper is to harness *by design* quantum tunneling as a computational resource and to use it as a controllable feature that can be engineered to provide additional processing power to a quantum algorithm. Furthermore, we have derived a detailed mathematical procedure to build a family of Hamiltonians to solve *K*-SAT instances via continuous quantum walks with potential barriers and quantum tunneling. Based on the structure of these Hamiltonians, we have used degenerate perturbation theory to derive an analytical approximation of the probability of finding satisfying assignments to *K*-SAT instances under our algorithmic approach.

As for the set of parameters required to run our algorithm, we have made an extensive numerical study that has allowed us to present good estimates of those parameters. It remains as future work to derive analytical formulae for this set of parameters, a future work which is encouraged by our numerical results.
